# Editorial: Neuro-Development and Psychological Issues in Congenital Heart Defects

**DOI:** 10.3389/fped.2017.00297

**Published:** 2018-01-12

**Authors:** Antonio F. Corno, Elisabeth M. W. J. Utens

**Affiliations:** ^1^Cardiovascular Research Center, University of Leicester, Leicester, United Kingdom; ^2^Glenfield Hospital, Leicester, United Kingdom; ^3^ErasmusMC-Sophia, Department of Child and Adolescent Psychiatry/Psychology, Rotterdam, Netherlands; ^4^Research Institute of Child Development and Education, University of Amsterdam, Amsterdam, Netherlands; ^5^Department Child and Adolescent Psychiatry, Academic Center for Child Psychiatry the Bascule, Academic Medical Center, Amsterdam, Netherlands

**Keywords:** congenital heart defects, neurodevelopment, psychology, surgery, neurodevelopmental functioning, cardiac surgery

**Editorial on the Research Topic**

**Neuro-Development and Psychological Issues in Congenital Heart Defects**

The operative mortality in infants with congenital heart defects has dramatically decreased in the past few decades to less than 3% in all databases of Europe and North America. As a result, attention has been moved from the survival to the quality of life. Surviving children often experience neurodevelopmental deficits and behavioral, emotional, and social problems, which can have a profound impact on their quality of life. This Research Topic focused on recent studies conducted in this field, to predict, evaluate, and manage the neurodevelopmental and psychological outcomes after congenital heart surgery.

Moon et al. studied 180 adolescents, analyzing the relationship between parental rearing behavior, resilience, and depressive symptoms. They demonstrated that parental rearing behaviors, such as emotional warmth, rejection, punishment, control, and overprotection have a significant influence on the resilience of the adolescents. They suggested that parenting attitudes, gender, age, and severity of the defects should be taken into consideration when developing intervention programs to increase the resilience and reduce the depression in these adolescents.

In another study on children and adolescents with congenital heart defects, Meentken et al. did an extensive review of post-traumatic stress, with a particular focus on stress related to medical interventions and treatment due to their underlying congenital heart defects, particularly for invasive interventions. The authors concluded that children with congenital heart defects present with an elevated risk of developing posttraumatic stress. Therefore, early screening of psychological problems and, if indicated, referral for psychological treatment should be made early on in this group of patients.

Pike et al. evaluated memory deficits in 80 adolescents and young adults with congenital heart defects, more than a decade following their last surgery in comparison to 76 healthy controls. Long after surgery, the group with congenital heart defects demonstrated significant verbal, attention, and working memory deficits over the control group. To enhance patient memory/self-care, the authors recommend to reduce anxiety, improve self-efficacy, and use of visual patient education material.

Buratti et al. studied 184 children, adolescents, and their parents, where heart malformations were divided in mild, moderate, and severe. Irrespective of the severity of the cardiac malformation, a strong association was found between the parent’s ratings of cognitive problems and the children’s and adolescents’ results on intelligence (Wechsler) scales, with this association present for all ages.

Helm et al. conducted a survey into transition in Germany in 1828 patients with congenital heart defects after they had turned 18 years of age. Their survey revealed that after age 18, many young adult patients had not been transferred to certified adult congenital heart defects providers and about 1/3 were not in continuous care at a specific adult congenital heart defects clinic/heart center. These observations regarding the adult certification were particularly disappointing and indicative of a large information gap and inadequate education in the current clinical practice.

Kolaitis et al. performed an extensive literature review into parental mental health at the time of the diagnosis of congenital heart defect, following cardiac surgery, and at long-term, assessing the need for psychological care. Their review confirmed that parents of children with congenital heart defects, and especially the mothers, are at higher risk for a variety of mental health problems at all different time periods of their children’s illness.

In a literature review, Hövels-Gürich identified that the factors influencing the neurodevelopment in infants undergoing cardiac surgery are not only related to the methods of cardiopulmonary bypass, procedure specific risk factors, and postoperative management but also patient specific risk factors, family and environmental factors.

Ryberg et al. investigated 228 children who underwent either surgery or interventional cardiology procedure. Their research demonstrated that socioeconomic status of the families and the severity of diagnosis had a significant influence on the full scale IQ of the children.

Kasmi et al. focused on neurodevelopment and psychiatric outcomes in a specific group of patients: those born with transposition of the great arteries. The authors conducted a detailed systematic review. They describe the results within a life-span perspective, putting particular emphasis on adolescent/young adult neuropsychological outcomes, describing potential mechanisms by which pediatric neurodevelopmental impairments can have negative influences into adulthood and also interventions to improve the clinical outcomes.

In response to an increased need for patient information congenital heart defects, Etnel et al. designed a pilot project developing an online, evidence-based information portal, with information on aortic and pulmonary valve disease, supported by both patients and physicians. If successful, this information portal will be further developed and expanded to include all common congenital heart defects, translated into other languages, and developing into a public information portal to serve patients’ relatives and the general public at large.

The studies and reviews collected in this Research Topic demonstrate that the success obtained by substantially reducing the mortality in the repair of congenital heart defects has not been followed by corresponding improvement in the quality of life.

While surgery does provide patients with a better life style related to their physical health, with less cyanosis, heart failure, and better exercise tolerance, much progress is still needed to improve the neurodevelopment outcomes and psychological health of these patients. We are now beginning a new era of research and clinical efforts to prevent and reduce the negative impact of congenital heart defects. Improved psychosocial interventions that caregivers, social workers, and behavior health providers can deliver to support patients and their families are urgently needed.

## Author Contributions

AC and EU co-wrote this manuscript.

## Conflict of Interest Statement

The authors declare that the research was conducted in the absence of any commercial or financial relationships that could be construed as a potential conflict of interest.

